# Comparison of classical methods for bone age determination with Capitohamate planimetry on wrist X-rays

**DOI:** 10.55730/1300-0144.5917

**Published:** 2024-03-11

**Authors:** Hasan YILDIZ, Nadire ÜNVER DOĞAN, Mehmet ÖZTÜRK, Zeliha FAZLIOĞULLARI, Muslu Kazım KÖREZ, Ahmet Kağan KARABULUT

**Affiliations:** 1 Department of Anatomy, Faculty of Medicine, Selçuk University, Konya, Turkiye; 2 Department of Radiology, Faculty of Medicine, Selçuk University, Konya, Turkiye; 3 Department of Biostatistics, Faculty of Medicine, Selçuk University, Konya, Turkiye

**Keywords:** Age determination, Greulich-Pyle, Tanner-Whitehouse, skeleton, carpal bones

## Abstract

**Background/aim:**

This study aims to compare the Greulich–Pyle (GP) and Tanner–Whitehouse 2 (TW2) methods, both used in left wrist radiography for bone age determination in the pediatric age group, with the Capitohamatum method (CH) due to the importance of determining bone age in the pediatric period.

**Materials and methods:**

Direct radiographs of 210 female and 291 male individuals between the ages of 0–15 without any pathology in the left wrist bones were obtained; 501 os capitatum and os hamatum on anterior-to-posterior direct graphic images were measured using the GP, TW2, and CH planimetry methods. The estimated age of each measurement was calculated by evaluating the relationship between chronological age and sex.

**Results:**

In male individuals, it was determined that the estimates obtained using the GP method were, on average, 5.1 units lower than the actual ages; the estimates obtained with the TW2 method were, on average, 1.7 units higher than the actual ages. In the female individuals, age estimations obtained by the GP and TW2 methods were 1.4 and 0.5 units lower, respectively, than the chronological ages. It was determined that the ages estimated by the CH method were almost the same as the chronological ages, with no deviation in the estimation method.

**Conclusion:**

According to the study findings, it was concluded that the CH method can be used reliably with high accuracy for chronological age determination of children as an alternative estimation method to the GP and TW2 atlases.

## 1. Introduction

Determining age by bone maturation is a commonly used procedure in Türkiye. Bone age determination is important for various metabolic disorders, nutrition, and endocrine disorders—and for criminal liability and other legal purposes in judicial cases [1]. For example, medically accurate age determination can help diagnose endocrine disorders and be useful in following up with patients receiving hormone therapy. It is also useful when deciding on the correct surgical intervention in orthopedics [2].

Bone age is one of the biological indicators of maturity used in clinical practice and is a critical parameter of a child’s medical assessment [20]. Radiologic evaluation of bones and its adaptation to present atlases are essential as this method is the most commonly used, and real-like values are obtained in age determination in clinical practice. The standards based on the method of detecting ossification points with epiphyseal and diaphyseal lines and detection of the periods of growth plate maturation in bones are used in this method. The hand and wrist are the most preferred sites for radiographic assessment in studying bone age and the most suitable areas with the required conditions to significantly assess radiographies. Skeletal maturity is assessed using the hand and wrist X-rays. This assessment is based on comparing ossification and maturity of hand and wrist epiphyses with the radiographs of individuals by using atlases and methods formed according to current standards [3]. The hand and wrist have recently been used to detect skeletal maturity periods in the growth process. They are the most suitable sites with the conditions required for effective radiographic assessment. Radiologists analyze skeletal maturation using hand and wrist X-rays. The most commonly preferred methods for bone age determination in hand-wrist radiographs are the Greulich–Pyle (GP) and Tanner–Whitehouse 2 (TW2) methods [4,5]. Although GP is one of the most widely used bone age estimation methods, its most significant disadvantages are its high inter- and interrater variability, the fact that it does not apply to some specific populations, and the last available version is from 1959 [3,5,20]. The biggest advantage of the TW2 method is that it assigns a digital score to each stage of maturation of the hand and wrist bones and allows the live expression of the sum of these scores when viewing maturity. The Gök atlas is used in Türkiye; it was created by Şemsi Gök and colleagues in 1985 by adapting the GP atlas and is used frequently by forensic experts in the country [21]. In Büken et al.’s study on the Gök atlas, which includes the 11–22 age group, the difference between chronological age and bone age according to the male and female age groups was more than one year between the ages of 15–19 in men and between 11–18 in women [19]. However, the possibility of making different estimates due to some evaluation errors during radiographic examination for age determination exists. Since direct radiography is a 2-dimensional reflection of a 3-dimensional object, any angle error in the position of the area radiographed may lead to incorrect evaluation of the bones of that region, which has been stated as its biggest disadvantage [15,16].

Our study aims to present the comparative effectiveness of single and multiple assessment methods and detect which was more reliable and applicable by applying the GP, TW2, and CH methods to female and male children in Konya (Türkiye). As a result, the study aims to compare the GP and TW2 methods used in left wrist radiography for bone age determination in the pediatric age group with the CH due to the importance of determining bone age during the pediatric period.

## 2. Materials and methods

Direct anterior-to-posterior (AP) hand-wrist radiographs stored for the last five years in the PACS archive of the Department of Radiology (Selçuk University) were retrospectively used in this study. The Os capitatum and os hamatum sites of 501 patients aged 0–15 were measured on direct AP radiographs. The correlation of these measurements with the GP method, the TW2 method, age, sex, and chronological age was retrospectively assessed. Technically improper images or those including pathologies such as a metabolic disorder and fractures were not included. The number of hand-wrist radiographs evaluated according to age and sex are shown in Table 1.

The chronological age of each patient was calculated using the duration between the date of birth and the date the radiograph was taken. The bone ages of the patients were then calculated according to the TW2 and GP methods for all cases. Twenty bones in the hand and wrist were assessed one by one according to their stages of maturation on the radiographs using the TW2 method. The maturation stage of the assessed bones was thus determined. Each bone received a score according to its maturation stage, and separate tables were created for the total scores of female and male individuals. The TW2 values used in our study were calculated for all cases, and the most commonly accepted TW2 scores were retained. However, bone age is not calculated monthly with this method but expressed in decimals. For example, in a result calculated as 12.5, the place after the decimal point is equal to 5/10 of the year, which means this particular patient is 12 years and 6 months old, according to the TW2.

In the GP method, the radiograph was matched with the present radiographic image according to female and male individuals in the atlas for each patient. Each image depicts a separate standard for each sex, and the bone age corresponding to the standard is given in months and years. This study considered the older and more current images of each case, and the most suitable ones were matched.

**Figure 1:**
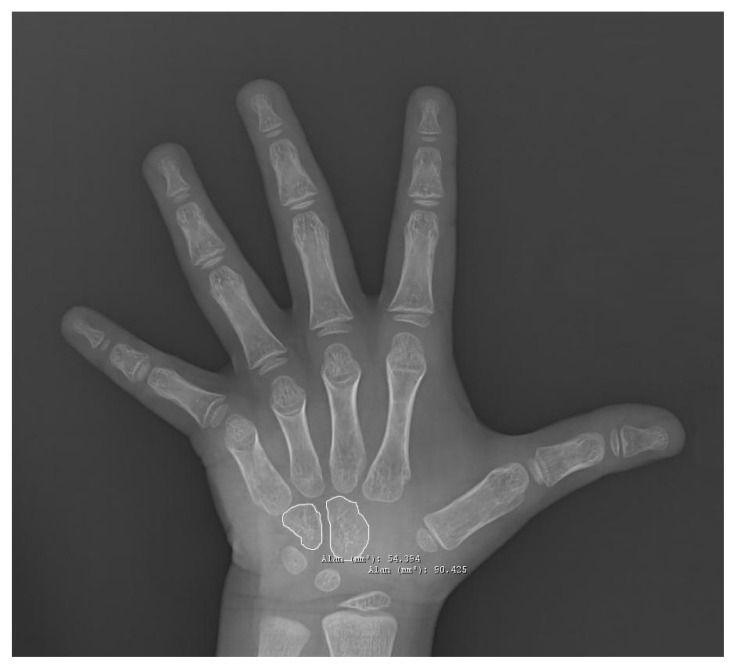
Os capitatum and os hamatum measurements according to the Capitohamatum planimetry method.

Os capitatum and os hamatum radiography areas were measured using the CH method (Figure 1). These two areas were calculated by combining the CH site for each hand-wrist area.

### 2.1. Statistical method

All data were digitalized, and statistical analysis was performed with *R* version 3.6.0 (The *R* Foundation for Statistical Computing, Vienna, Austria; https://www.r-project.org) software program. The normality of the data was assessed using the Anderson–Darling Normality test and Q–Q plots. Linear and polynomial (quadratic) regression equations were set up to predict the chronological ages of the individuals with the areas measured using the Capitohamate (CH) planimetry method. Whether one of these two regression models was more successful than the other in predicting chronological age was determined with model fit measures (*R*^2^: coefficient of determination; root mean square error (RMSE); model selection criteria (Akaike Information Criteria (AIC); and Bayesian Information Criteria (BIC)). The model with a high *R^2^* and a low RMSE value among model fit measures and low AIC and BIC values among the model selection criteria was selected as the correlated model for predicting chronological age (quadratic regression model). Chronological age predictions of both males and females were performed with a 95% confidence level with the help of selected quadratic regression equations. In addition, chronological ages predicted using the GP, TW2, and CH methods and the real ages of the individuals were compared using Friedman’s test and, later, with the Bonferroni-corrected Durbin–Conover post-hoc test. The relationships between the patients’ real ages and chronological ages predicted with all methods were assessed with Spearman’s *rho* correlation analysis. The agreement between the methods was assessed using the Bland–Altman (B–A) method, the intraclass correlation coefficient, the concordance correlation coefficient, and precision and accuracy values.

## 3. Results

**Figure 2:**
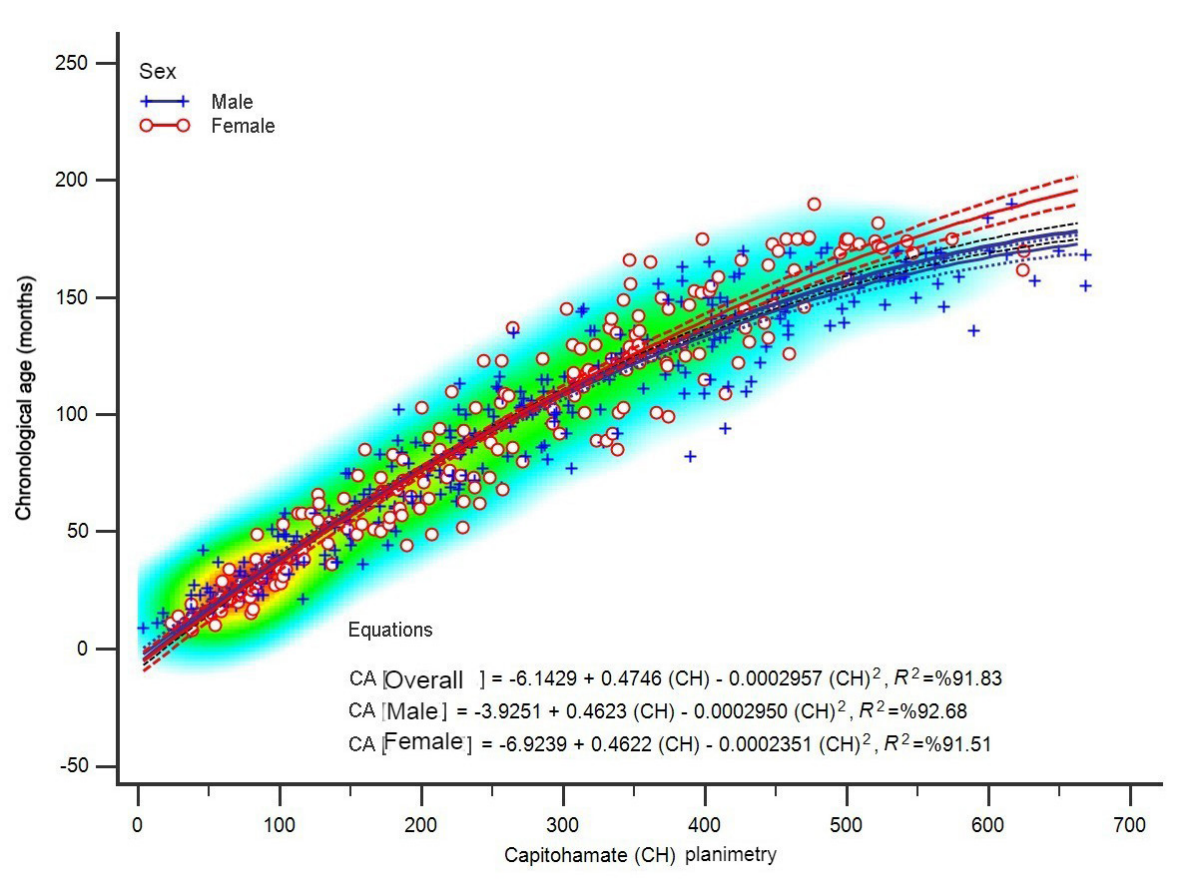
General quadratic regression curves according to sex.

The regression models used to predict the chronological age based on CH areas are given in Table 2. According to the results obtained in Table 2, both linear and polynomial regression models can significantly be used for predicting chronological age; however, it was observed that the quadratic regression model was more efficient at data modeling according to the fit and selection criteria than the linear method. The regression coefficients related to the prediction equation obtained using the quadratic regression model and the set-up model were significant, and the coefficient of determination rate was above 90% for both male and female children. According to this data, the equations for chronological age prediction combining the sexes and for each sex separately are as follows (Figure 2):

The equation used for combined male/female data:


$$\text{Chronological age }(\text{month})=-6.1429+0.4746\times (\text{CH})-0.0002957\times {\left(\text{CH}\right)}^{2},R^{2}=92.68\%$$

For males:


$$\text{Chronological age }(\text{month})=-3.9251+0.4623\times (\text{CH})-0.0002950\times {\left(\text{CH}\right)}^{2},R^{2}=92.68\%$$

For females:


$$\text{Chronological age }(\text{month})=-6.9239+0.4622\times (\text{CH})-0.0002351\times {\left(\text{CH}\right)}^{2},R^{2}=91.51\%$$

The differences between the real and chronological ages predicted with different prediction methods for each sex were assessed using Friedman’s test and, subsequently, the Bonferroni-corrected Durbin–Conover test for multiple comparisons (Table 3).

**Figure 3:**
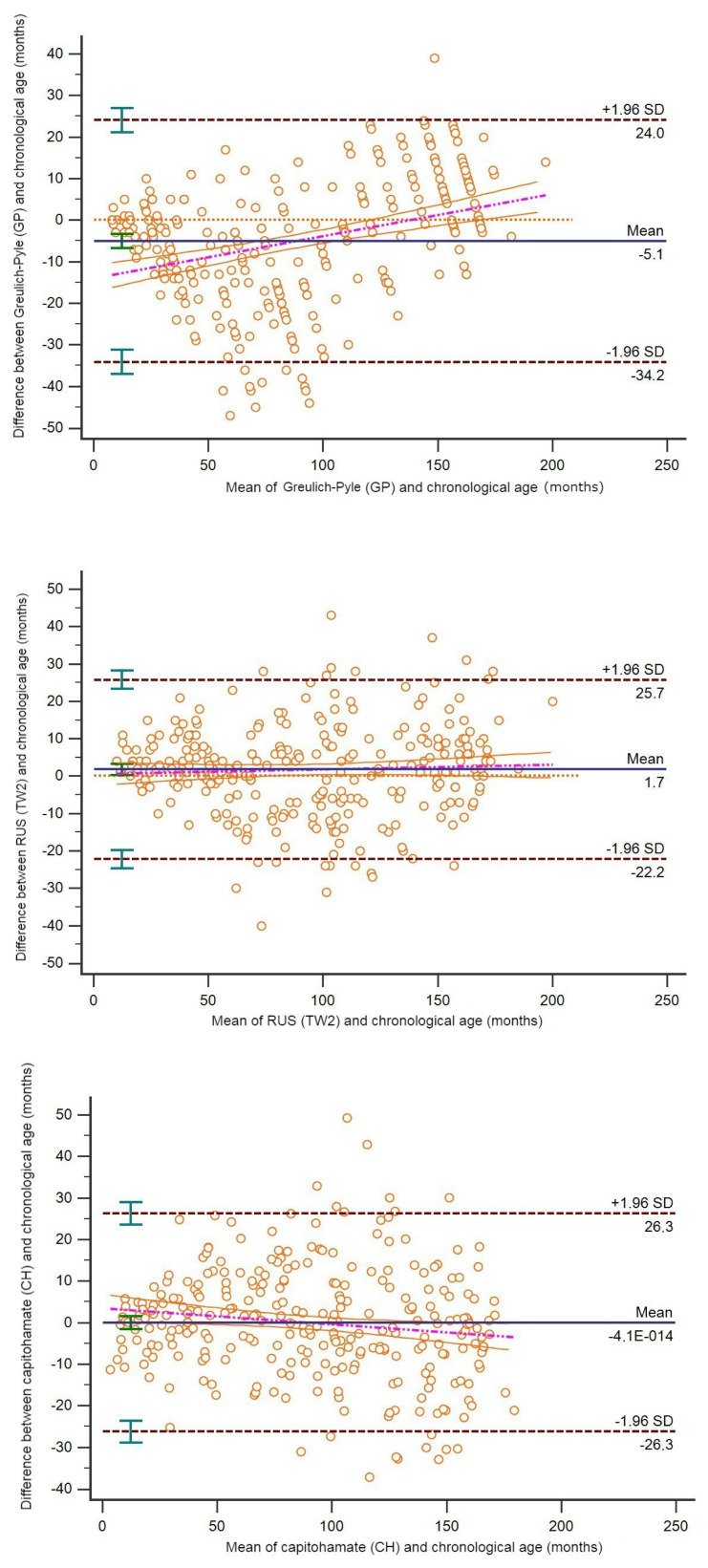
Bland–Altman plot revealing the correlation between chronological age (month) and the age prediction methods in male children.

The agreement and correlations between the real ages of the individuals and age predictions of the methods used for chronological age prediction were assessed and are shown in Table 4. All methods corresponded and correlated to determine the real age. However, for males, the predictions obtained with the GP method (mean age: 85.57 ± 54.68) were a mean 5.1 units lower than the real ages. The predictions obtained using the TW2 method (mean age: 94.08 ± 49.18) were mean 1.7 units higher than the real ages determined with the B–A method (Figure 3). The ages predicted with the CH method (mean age: 90.64 ± 47.65) were almost the same as the chronological ages (mean age: 90.64 ± 49.50), with no deviation in the prediction method (Figure 3).

**Figure 4:**
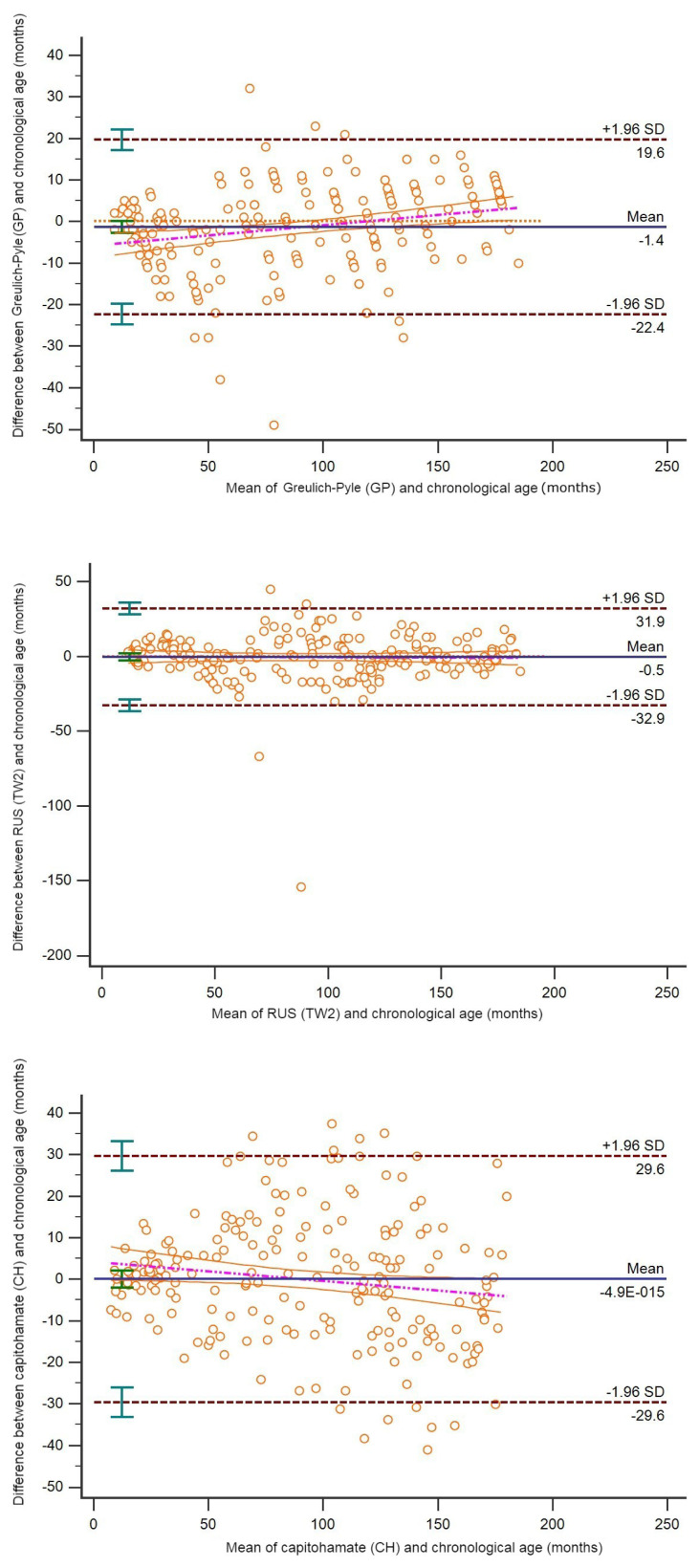
Bland–Altman plot revealing the correlation between chronological age (month) and the age prediction methods in female children.

For females, the ages predicted with both the GP (mean age: 90.21 ± 54.49) and TW2 (mean age: 91.86 ± 51.24) methods were a mean 1.4 and 0.5 units lower than the chronological ages (mean age: 91.58 ± 51.89), respectively. No deviation in the CH method was found (mean age: 91.58 ± 49.63) (Figure 4).

According to the results, there was a statistically significant difference between the real ages and chronological ages predicted with the GP and TW2 methods in male children; however, no statistically significant difference existed between the real ages and chronological ages predicted using the CH method. The ages predicted with the GP method were lower than the real ages of children; additionally, the ages predicted with the TW2 method were higher than the real ages. However, the ages predicted with the GP and TW2 methods significantly differed from those predicted with the CH method.

For female children, there was no significant difference between their real ages and the predicted ages obtained with any of the methods; in addition, the ages predicted using the CH method (mean age: 91.58 ± 49.63) were different from those predicted with the GP method.

According to these results, it was concluded that the CH method is reliable and has a high accuracy for chronological age determination in children; it can also serve as an alternative prediction method for the GP and TW atlases.

## 4. Discussion

The most crucial finding in this study was that the CH method is reliable and has high accuracy for chronological age determination in children; it can also be a prediction method alternative to the GP and TW atlases. These methods are accepted as valid scientific methods for legal purposes by courts worldwide [12]. Histological, morphological, and radiological methods are used in age determination [13]. In a study conducted on 515 obese children in Brazil, the bone age was found to be older than the chronological age according to the GP atlas in all groups [17]. Kemperdick applied the GP method in Germany and reported that it could be used for children living in West Germany if correction tables were included [18].

Buken et al. investigated whether the GP method was appropriate in estimating the forensic age of Turkish children, and, as a result, the standard deviation was found to be more than one year for girls between the ages of 12 and 15 and boys at 12, 15, and 18 years of age. However, it was not known at the time whether other methods were more useful than this method. The authors concluded that the GP method should be used cautiously in cases of possible criminal liability in forensic age diagnosis unless another method is more useful [19].

In a study that included 303 male and 122 female patients between 2009 and 2010 in Iran, both sexes were divided into three subgroups (6–10, 10–14, and 14–18). The GP atlas was acceptably accurate and applicable in Iranian female children, considering that the bone age of female participants was 0.5 months higher [7].

A meta-analysis published in 2019 assessed whether the GP atlas could be applied to all ethnic groups, and a total of 49 studies between 1950 and 2017 were included in the assessment; 35 studies correlated with the meta-analysis. As a result of this analysis, no significant difference between bone age and chronological age in African male cases, Asian female cases, Caucasians, and South American individuals was found; however, it was emphasized that the GP atlas should be carefully used when applied to Asian male and African female patients [6]. In addition, while the correlation of these studies was being assessed, i.e. whether the individuals had any diseases, a difference was found between the mean chronological age and mean bone age, and only studies written in English were determined as the selection criteria.

Patients aged between 10 and 22 were included in a study performed in Ethiopia in 2015, and bone age was found to be 8.7 months lower in males and 11.8 months lower in females compared to the chronological age in the GP atlas; the results were not statistically significant, and the authors stated that new methods must be developed in the future [8]. Only the 0–15 age range was assessed in our study, which was different from the study mentioned above; in our research, the GP atlas was mean 5.1 months lower in the male group between the ages of 0–15 and mean 1.4 months lower in the female group in the same age category compared to chronological age.

In a study by Malina et al. comparing the TW2 and TW3 atlases in 1831 with young footballers aged between 10 and 17 in 2018, the ages of the participants were 0.97 and 1.16 years delayed compared to the chronological age according to TW2 and TW3 atlases. While 42% of the players that were classified as average according to the TW2 atlas were delayed according to the age range compared with the TW3 atlas, 64% of the patients who developed early according to the TW2 atlas were considered average compared to the TW3 atlas. The authors stated that the GP and TW3 atlases could be initial choices for clinical use compared to the TW2 [9].

In our study, the GP method measured the age range lower, and the TW2 method measured the age range higher in male children and lower in female children. Different from this study [9], we believe that the CH planimetry method is more convenient for current populations than other methods. In a study conducted on 611 children in Taiwan, while the GP atlas measured the age as 1.24 years higher in female children, it was 0.61 years lower in male children compared to chronological age [10]. According to a review assessing the GP method applied to 33 female and 37 male participants in the Eastern Uttar Pradesh region of India, age retardation was higher in males than females. The authors stated that a larger population was required to apply GP [14].

As seen in the GP atlas in a study by Choi et al. on 391 Korean children in 2018, the probability of an earlier appearance of os capitatum and os hamatum nuclei was higher in female children than in male children. In addition, the authors found a strong positive correlation between chronological age and CH planimetry measurement. They also indicated rising slopes in the planimetry curves of female and male capitatum and hamatum samples. The most significant correlations were between os triquetrum, capitatum, and hamatum bones present in all 20 hands. The mean bone age value predicted with the GP method was lower than that measured with the CH planimetry method (p < 0.0001). The 95% confidence interval range was between −10.5 and 13.4 months in age prediction using the CH planimetry method and between −21.1 and 29.5 months using the CP method. While the age was found to be 1.4 months higher using the CH method, it was −4.2 months lower when employing the GP method [11].

In our study conducted on 501 children, the GP method was −5.1 months lower, the TW2 was 1.7 months higher, and the CH method was 0 months in male children; in addition, the GP method was −1.7 months lower, the TW2 was 0.5 months higher, and the CH method was 0 in female children. This study used the largest number of subjects among other studies using the GP method.

## 5.Conclusion

In this study, we performed analyses on 501 children by expanding on Choi et al.’s study on 391 children; a statistically significant difference was found between male children’s chronological ages and the chronological ages predicted using the GP and TW2 methods; however, no statistically significant difference existed between the children’s real ages and chronological ages predicted with CH. The ages predicted with the GP method were lower than the real ages of the children, and the ages predicted with TW2 were higher than the children’s real ages. However, the ages predicted with GP and TW2 significantly differed from those predicted with CH.

For female children, no significant difference could be found between their real ages and ages predicted with any of the observed methods; however, the ages predicted with the CH method differed from those obtained with GP.

In conclusion, the CH planimetry method was useful for bone age assessment of individuals observed in Konya. Age prediction w ith a simple application method resulted in 91.83% reliability and, within less than one minute, led to an advantage over other methods. In addition, this is the first study performed in Türkiye and the only study conducted using the CH planimetry method with such a large number of subjects. The CH planimetry method can be performed automatically after ethnicity and sex parity, save money in the future if integrated into PACS, and lead to more precise bone age assessment.

## Figures and Tables

**Table 1:** Distribution of hand-wrist radiographs by age and sex.

Age (months)	Male (n = 291)	Female (n = 210)
1–11	10	6
12–23	22	18
24–35	16	20
36–47	27	8
48–59	17	17
60–71	20	15
72–83	23	11
84–95	20	14
96–107	22	12
108–119	22	13
120–131	12	24
132–143	20	20
144–155	21	12
156–167	24	9
168–179	13	19
180	2	2

**Table 2:** Significance statistics for regression models set up to predict chronological age based on CH area.

	Regression coefficients and model significances				Model fit measures			Model selection criteria	
Method	*t* *_β_*__0__	*t* *_β_*__2__	*t* *_β_*__2__	*F*	*r*	*R* ^2^	RMSE	AIC	BIC
Linear model									
General	7.99*	65.35*	-	4271.09*	0.946*	0.896	16.29	4215.65	4228.29
Male	8.18*	50.86*	-	2586.88*	0.949*	0.900	15.64	2423.91	2434.92
Female	2.51*	44.55*	-	1985.25*	0.951*	0.905	15.94	1764.92	1774.96
Quadratic model									
General	−2.32*	31.22*	−11.77*	2793.56*	0.958*	0.918	14.41	4094.88	4111.74
Male	−1.86*	25.81*	−10.27*	1815.61*	0.963*	0.927	13.37	2335.11	2349.78
Female	−2.09*	16.98*	−4.91*	1115.09*	0.957*	0.915	15.09	1743.73	1757.13

*t_β_*__0__, *t_β_*__1__, *t_β_*__2__: significance values for regression coefficients in the linear and quadratic regression models (showing stability and slope coefficients, respectively); *F*: significance value for models; *R*^2^: coefficient of determination; RMSE: root mean square error; AIC: Akaike information criteria; BIC: Bayesian information criteria;

* shows statistical significance (p < 0.05).

**Table 3:** Results of the comparison between chronological age and predicted age values obtained using different methods for each sex.

	Mean ± SD	Median (IQR = Q1–Q3)	p-value	Multiple comparison
Male			<0.001	
Chronological Age (month)	90.64 ± 49.50	90.50 (46–136)		GP–TW2
GP Age Prediction (month)	85.57 ± 54.68	72 (36–144)		CA–TW2–CH
TW2 Age Prediction (month)	94.08 ± 49.18	91.50 (51–140)		CA– GP–CH
CH Age Prediction (month)	90.64 ± 47.65	92.67 (51.71–134.49)		GP–TW2
Female			0.011	
Chronological Age (month)	91.58 ± 51.89	91 (49–131)		
GP Age Prediction (month)	90.21 ± 54.49	90 (36–132)		TW2–CH
TW2 Age Prediction (month)	91.86 ± 51.24	96.50 (40–134)		GP
CH Age Prediction (month)	91.58 ± 49.63	95.49 (43.80–130.69)		GP

CA: Chronological Age; GP: Greulich–Pyle method; TW2: Tanner–Whitehouse 2 method; CH: Capitohamate method; Mean ± SD: mean ± standard deviation; IQR (Q1–Q3): interquartile range (1^st^ quartile–3^rd^ quartile); p-value: calculated using Friedman’s test; Multiple comparison: Bonferroni-corrected Durbin–Conover post-hoc tests were used.

**Table 4:** Results on agreement and correlation statistics between chronological age and predicted age values obtained using different methods for each sex.

	CA & GP	CA & TW2	CA & CH
Male			
B–A method (95% LoA)	−5.1 (−34.2 to 24)	1.7 (−22.2 to 25.7)	0 (−26.3 to 26)
ICC (95% CI)	0.959 (0.949 – 0.967)	0.968 (0.960 – 0.975)	0.962 (0.952– 0.969)
CCC (95% CI)	0.954 (0.944– 0.963)	0.968 (0.959–0.974)	0.962 (0.952 – 0.970)
Precision	0.964	0.969	0.963
Accuracy	0.990	0.999	0.999
Spearman’s *rho* (95% CI)	0.971 (0.963–0.977)	0.968 (0.960–0.975)	0.962 (0.952–0.970)
Female			
B–A method (95% LoA)	−1.4 (−22.4 to 19.6)	−0.5 (−32.9 to 31.9)	0 (−29.6 to 29.6)
ICC (95% CI)	0.979 (0.973–0.984)	0.948 (0.932–0.960)	0.955 (0.942–0.966)
CCC (95% CI)	0.979 (0.973–0.984)	0.948 (0.932–0.960)	0.956 (0.942–0.965)
Precision	0.981	0.948	0.957
Accuracy	0.998	0.999	0.999
Spearman’s *rho* (95% CI)	0.981 (0.975–0.985)	0.949 (0.933–0.961)	0.956 (0.943–0.966)

B–A method (95% LoA): Bland–Altman method (95% Limits of Agreement); ICC: intraclass correlation coefficient, CCC: concordance correlation coefficient; 95% CI: 95% confidence interval; CA: chronological age; GP: Greulich–Pyle method; TW2: Tanner–Whitehouse 2 method; CH: Capitohamate method.

## Abbreviations

AIC: Akaike Information Criteria
BIC: Bayesian Information Criteria
CCC: concordance correlation coefficient
CH: Capitohamatum method
GP: Greulich–Pyle
ICC: intraclass correlation coefficient
*R* ^2^: coefficient of determination
RMSE: root mean square error
TW2: Tanner–Whitehouse 2
